# Lifestyle habits and type 2 diabetes traits in patients from healthcare centers in Dubai, United Arab Emirates: a cross-sectional study

**DOI:** 10.3389/fendo.2025.1436536

**Published:** 2025-05-06

**Authors:** Kenana Hatab, Fadila Serdarevic, Aisha Yousuf, Sarah Al Ali, Khawla Eissa Ahmed M. Al Hajaj, Fatima Mohamed Abdulla Mohamed Almarzooqi, Leena Tawfiq Swaidan, Elsheikh Farah ElHassan, Mahra Nooruddin Abdulwahid Kazim, Hanan Hamza Ahmed Abdalla, Hanan Ali Al-Muhaureq, Salah Mohammed Thabit, Hassan Abdulla BaKhamis, Sabina Semiz

**Affiliations:** ^1^ College of Medicine and Health Sciences, Khalifa University of Science and Technology, Abu Dhabi, United Arab Emirates; ^2^ Sarajevo School of Science and Technology, Sarajevo Medical School, Sarajevo, Bosnia and Herzegovina; ^3^ Erasmus Medical Center, Rotterdam, Netherlands; ^4^ Center for Epidemiology and Statistics ("EpiSTa"), Sarajevo, Bosnia and Herzegovina; ^5^ Za’abeel Health Center, Dubai Health, Dubai, United Arab Emirates; ^6^ Dubai Health Authority, Dubai, United Arab Emirates; ^7^ Almizhar Health Center, Dubai Health, Dubai, United Arab Emirates; ^8^ Al Towar Health Center, Dubai Health, Dubai, United Arab Emirates; ^9^ Al Barsha Health Center, Dubai Health, Dubai, United Arab Emirates; ^10^ Center for Biotechnology, Khalifa University of Science and Technology, Abu Dhabi, United Arab Emirates

**Keywords:** obesity, diabetes, diet, physical activity, gender

## Abstract

**Objectives:**

Given the increasing prevalence of type 2 diabetes (T2D) in the United Arab Emirates (UAE), in this cross-sectional study we analyzed the dietary habits, physical activity, as well as anthropometric, clinical, and laboratory parameters in patients with T2D and nondiabetic (ND) subjects from the UAE.

**Subjects and Methods:**

This study included 344 UAE nationals, age 18 or older, who completed a questionnaire to assess their dietary habits and physical activity. The mean age of the recruited participants was 50.9 years (+/-14.1) and females presented 63.4% of the sample size. The linear regression analysis was applied to analyze all clinical parameters and dietary habits as outcomes, while a logistic regression analysis was used to determine the association of patient status with physical activity, as an outcome. All models were controlled for age, gender, and BMI.

**Results:**

There was a difference in dietary habits between the groups (B=-3.7; CI -6.42, -0.92; p=0.009), where patients with T2D had a lower dietary score than ND subjects, indicating a healthier diet. Our results showed that 19% of T2D patients and 32% of nondiabetic individuals were concerned about their weight. Both groups considered dietary changes important, and more than 90% of participants expressed high or moderate confidence in making those changes. Furthermore, the male nondiabetic subjects demonstrated a 4 times higher level of physical activity versus their diabetic counterparts (OR=4.01, CI: 1.01, 15.9, p=0.04), while the difference in physical activity between female subjects with and without T2D was not demonstrated.

**Conclusions:**

Our results showed that patients with T2D consumed a healthier diet than nondiabetic subjects, probably resulting in more favorable total cholesterol and LDL-cholesterol profiles. A gender difference in physical activity was also observed in this sample of UAE population. Our findings suggest that although patients with T2D in the UAE may adopt healthier dietary habits, there remains a significant need to address physical activity disparities, particularly among women, to improve overall health outcomes in this population. Addressing these habits through targeted clinical interventions and lifestyle medicine approach can optimize long-term management and prevention strategies for type 2 diabetes in this region.

## Introduction

Diabetes is considered as one of the major pandemics of the 21^st^ century, affecting 537 million people in 2021 (1). This chronic metabolic disorder, characterized by elevated blood glucose levels, is one of leading causes of morbidity and mortality, with its prevalence expected to increase significantly. It is estimated that by 2045 the number of diabetic patients will increase to 700 million worldwide, with the second highest expected increase (86%) reaching 136 million diabetic individuals in the Middle Eastern and North African (MENA) region (1). Furthermore, it is suggested that, in particular, the Gulf countries, located within this region, have high risk factors for type 2 diabetes (T2D) (2), with a prevalence of about 16% in the United Arab Emirates (UAE) (1), estimated to reach 21% by 2030 (3, 4).

The development of T2D is multifactorial, with a strong association to lifestyle factors such as obesity, unhealthy dietary habits, and a sedentary lifestyle. The global rise in obesity rates (5–7), which has been particularly noticeable in the Gulf countries (2, 8–10), is contributing to the escalating prevalence of T2D. Obesity, a major risk factor for insulin resistance and T2D, has reached alarming levels in the UAE (11). Based on the recent UAE national data, 74% of adults in the UAE are classified as overweight, with 37% of them being obese (12). Furthermore, childhood obesity is an increasing concern (13–16), with recent estimates indicating that 17% of children in the UAE are classified as overweight or obese (17). This is particularly worrying given that children who are overweight or obese face up to 80% risk of remaining obese later in adulthood, thus increasing their lifetime risk of developing T2D (18).

In addition to obesity, lifestyle factors such as physical inactivity and poor dietary habits play critical roles in the development of T2D. Previous studies performed in the UAE population showed that an accentuated exposure to sedentary lifestyle characterized by limited physical activity, combined with an increased consumption of calorie-dense food (19, 20) increase risk for obesity (7), insulin resistance (21), impaired glucose tolerance and onset of T2D (22). Despite these concerns, there remains a dearth of comprehensive studies examining the relationship between lifestyle habits and T2D-related traits in the UAE population.

Considering the growing burden of T2D in the region, the primary objective of this study is to investigate the dietary habits, physical activity levels, and their associations with T2D-related traits in individuals in the UAE. The secondary objectives aim to explore if participants’ gender may influence the relationship between lifestyle habits and diabetes-related traits in patients with T2D and in nondiabetic subjects, as well as to assess participants’ views and attitudes towards their weight and diet. Understanding these associations is crucial for developing targeted public health interventions and promoting lifestyle modifications that could reduce the growing incidence of diabetes and help guide preventive and clinical strategies tailored to the UAE and the broader region.

## Subjects and methods

### Subjects

We conducted a cross-sectional study involving 344 subjects, including 218 patients with type 2 diabetes and 126 nondiabetic subjects, who were recruited consecutively from March 2021 to September 2022 at Zabeel, Al Mizhar, Al Towar, and Al Barsha primary healthcare centers in Dubai, United Arab Emirates. The STROBE (Strengthening the Reporting of Observational Studies in Epidemiology) checklist was used as reporting tool (23). All participants met the major inclusion criteria, which included being a UAE national, 18 years old or older, and providing signed consent. Patients with chronic gastrointestinal diseases, including chronic liver disease, cholelithiasis, chronic pancreatitis, inflammatory bowel disease and gastroduodenal ulcer, chronic kidney disease, endocrine disorders, infection and hormonal therapy were excluded. Demographic data, anthropometric measurements, and biochemical parameters were obtained from the participant’s medical records.

The study was approved by the Research Ethics Committee of the Khalifa University, Department of Health Abu Dhabi and Dubai Scientific Research Ethics Committee, Dubai Health Authority.

### Questionnaire

In order to assess the participants’ dietary habits and physical activity, the study participants completed a questionnaire including the selected questions from the UK Diabetes & Diet Questionnaire (UKDDQ) and the Nurse Health Studies II Physical Activity questionnaire (NHSPAQ). Sixteen out of the nineteen questions contributed to the overall dietary score, while the remaining three questions addressed weight concerns, the significance of dietary changes, and the participants’ confidence in making such changes. The interviewed subjects were also asked to specify how frequently they consumed various food items from different groups over the past month. They were given options to describe their consumption frequency, ranging from “never,” “very rarely or less than once a week,” “1–4 times a week,” and “5 times to every day in a week.” The total dietary score was obtained by summing the raw values of all UKDDQ items, with higher scores indicating less healthy dietary choices (24). The Nurse Health Studies II Physical Activity questionnaire included questions about the participants’ physical lifestyle habits, classifying them as physically active based on their exercise duration and work type (25). This questionnaire included inquiries about the frequency of the participants’ physical activity per week in the past year and their participation in recreational activities. Additionally, the nature of the participants’ occupations contributed to the final evaluation, mainly if they were engaged in physically demanding fields. The hours of physical activity were systematically calculated and subsequently categorized into two distinct groups based on WHO recommendation (World Health Organization, Guidelines on physical activity and sedentary behavior, 2020) (26). Patients who exercised for 150–300 minutes or more per week were classified as exhibiting a “physically active” status, whereas patients engaging in less exercise in one week were classified as demonstrating a “physically inactive” status.

### Clinical and biochemical parameters

After obtaining consent, the study participants provided a blood sample for measuring various biochemical parameters, including but not limited to fasting glucose (FG), glycated hemoglobin (HbA1c), fasting insulin (FI), total cholesterol (TC), triglycerides (TG), high-density lipoprotein (HDL) cholesterol, low-density lipoprotein (LDL) cholesterol, and C-reactive protein (CRP). The body mass index (BMI) was calculated by dividing the participant’s weight by their height in square meters. BMI categories were defined according to the World Health Organization (WHO) classifications as underweight (BMI < 18.8 m^2), average weight (18.5-24.9 kg/m^2^), overweight (25-29.9 kg/m^2^), and obese (≥ 30 kg/m^2^). Homeostatic Model Assessment of Insulin Resistance (HOMA-IR) was calculated by using the equation HOMA-IR = [fasting insulin (pmol/l) x fasting glucose (mmol/l)]/156.26.

### Statistical analysis

We estimated the differences between diabetic and nondiabetic participants regarding their physical activity by using the logistic regression analysis, as well as regarding their dietary habits and biochemical parameters by using the linear regression analysis. We adjusted all analysis for age, gender, and BMI. Statistical significance was considered when the two-tailed p-value was < 0.05. Laboratory data were standardized using z-scores.

### Sensitivity analysis

We additionally adjusted all models for participants’ education.

## Results

The characteristics of the study participants are presented in **Table 1**. Out of all participants with type 2 diabetes, 60.7% were females and 66.1% completed high school or less, while in the group of nondiabetic subjects, 69.7% were females and 44% completed high school or less. Patients with T2D had on average BMI 30.4 (SD=6.3) and nondiabetic subjects had on average BMI 29.1 (SD=6.1), and based on the WHO classification, were considered as obese and overweight, respectively.

**Table 1:** Characteristics of patients with T2D and nondiabetic subjects participating in this study (n=344).

Characteristics	T2D patients (n=218)	Nondiabetic subjects (n=126)
Age, years	56.3 ± 12.1	41.3 ± 12.5
Gender, female %	60.7	69.7
BMI, kg/m³	30.4 ± 6.3	29.1 ± 6.1
Systolic BP, mmHg	129.4 ± 15.4	121.1 ± 15.4
Diastolic BP, mmHg	74.4 ± 10.9	75.4 ± 10.9
Fasting glucose, mmol/L	8.0 ± 3.0	5.6 ± 1.2
Fasting insulin, pmol/L	100.7 ± 98.9	72.3 ± 38.2
HOMA-IR	5.7 ± 12.2	2.6 ± 1.5
HbA1c, %	7.0 ± 1.5	5.5 ± 1.1
HbA1c, mmol/mol	53.3 ± 16.7	36.2 ± 8.3
Total cholesterol, mmol/L	9.8 ± 2.4	11.5 ± 2.4
HDL-cholesterol, mmol/L	2.9 ± 0.9	3.2 ± 0.8
LDL-cholesterol, mmol/L	5.6 ± 2.1	7.1 ± 2.2
Triglycerides, mmol/L	6.8 ± 3.1	5.4 ± 2.2
CRP, mg/dL	11.2 ± 31.6	5.2 ± 9.5
Physical activity, inactive %	82.2	72.7
Dietary score	37.7 ± 8.0	43.5 ± 9.2
Marital status, single %	17.7	36.8
Education, low %	66.1	44.0

The numbers are means (SD) for variables with normal distribution, median (IQR) for not normally distributed variables, and percentages for categorical variables.

HOMA-IR, Homeostatic Model Assessment for Insulin Resistance; HbA1c, hemoglobin A1c; HDL, high-density lipoprotein cholesterol; LDL, low-density lipoprotein cholesterol; CRP, C-reactive protein; BMI, body mass index.

As expected, the levels of fasting glucose, fasting insulin, HbA1c, HOMA-IR, triglycerides, and CRP were increased in patients with T2D, as compared to nondiabetic subjects, after adjusting for age, gender, and BMI (**Table 2**). Furthermore, HDL-cholesterol levels were lower in T2D patients. The concentration of TC and LDL-cholesterol was also lower in diabetic patients than in nondiabetic subjects (**Table 2**).

**Table 2:** The clinical and biochemical parameters in T2D patients and nondiabetic subjects.

	Model I*			Model II**		
Parameters	B	CI	p-value	B	CI	p-value
Systolic, mmHg	2.2	(-1.7, 6.0)	.3	.8	(-2.9, 4.6)	.7
Diastolic, mmHg	-.027	(-.09, 0.04)	.4	-1.4	(-3.0,.06)	**.06**
*BMI*	2.0	(.3, 3.6)	**.02**	–	–	–
Fasting glucose, mmol/L	1.2	(.8, 1.6)	**<.001**	1.1	(0.8, 1.5)	**<.001**
Fasting insulin, pmol/L	10.3	(-5.7, 26.3)	.2	6.1	(-9.6, 21.9)	.4
HOMA-IR	1.0	(-.9, 2.9)	.3	.6	(-1.3, 2.5)	.5
HbA1c, mmol/mol	9.4	(7.4, 11.4)	**<.001**	8.9	(6.9, 10.9)	**<.001**
Total cholesterol, mmol/L	-14.6	(-20.3,-8.9)	**<.001**	-14.7	(-20.5, -8.9)	**<.001**
Triglyceride, mmol/L	12.0	(5.2, 18.7)	**<.001**	11.5	(4.6, 18.4)	**.001**
HDL-cholesterol, mmol/L	-3.6	(-5.6, -1.6)	**<.001**	-4.5	(-6.3, -2.7)	**<.001**
LDL-cholesterol, mmol/L	-12.7	(-17.8, -7.7)	**<.001**	-12.8	(-18.0, -7.6)	**<.001**
CRP, mg/dL	2.2	(-2.1, 6.4)	.3	1.8	(-2.6, 6.3)	.4

Exposure: patient status * Linear regression analysis upon adjustment for age and gender ** Linear regression analysis upon adjustment for age, gender, and BMI.

HOMA-IR, Homeostatic Model Assessment for Insulin Resistance; HbA1c, hemoglobin A1c; HDL, high-density lipoprotein cholesterol; LDL, low-density lipoprotein cholesterol; CRP, C-reactive protein; BMI, body mass index. The bold values highlight significant differences between specific clinical and biochemical parameters presented in this table.

Most of the participants with T2D (82.2%) and nondiabetic subjects (72.7%) reported to be physically inactive (**Table 1**). We did not observe a difference in physical activity between T2D patients and nondiabetic subjects (OR=1.0 CI: 0.48, 2.43, p=0.85). Furthermore, our results also showed that males exercised 3.1 times more than female subjects upon controlling for patient status, age and BMI (OR=3.1, CI:1.58, 6.09, p<.001). We did not observe a significant difference in physical activity between the two groups of female subjects. The male subjects with T2D demonstrated a 4 times lower level of physical activity versus their nondiabetic counterparts (OR=4.01, CI:1.01, 15.9, p=0.04).

We observed a significant difference in dietary habits between patients with T2D and nondiabetic subjects (B=-3.8; CI -6.03, -0.72; p=0.01), indicating healthier diet consumption by T2D patients. Patients with T2D were more likely to consume less fast food and sweets, and more fruits and vegetables when compared to nondiabetic individuals (B=-1.8; CI -3.4, -0.2; p=0.029). Female participants with diabetes have a lower mean dietary score, indicating healthier dietary habits as compared to female nondiabetic subjects (B=-3.7; CI -6.40, -0.94; p=0.009). A similar trend in dietary habits between male participants with and without type 2 diabetes was also observed, however this difference was not significant (B=-2.5; CI -8.61, 3.65; p=0.42).

Due to observed significant interactions of gender with BMI upon age adjustment (p=0.02) and a tendency of gender interaction with diastolic blood pressure (p=0.06) (**Table 2**), we analyzed the BMI separately in female and male participants. As shown in the **Supplementary Table 1**, there was no significant difference in BMI and blood pressure values between male subjects with T2D and nondiabetic males. However, our results demonstrated a difference in BMI between female diabetic patients and nondiabetic subjects upon age adjustment (B=2.25; CI: -8.61, -3.65, p = 0.04) (**Supplementary Table 2**).

The participants’ views regarding their weight and diet change are summarized in **Table 3**. These findings indicate that about 19% of T2D patients and 32% of nondiabetic subjects were moderately or very concerned about their weight. Changing their diet was shown to be of high or moderate importance to both of these study groups. Furthermore, both diabetic and nondiabetic subjects showed a self-confidence in diet change, with about 96% of T2D patients and 92% nondiabetic individuals demonstrating high or moderate confidence in changing their diet (**Table 3**). There was no significant difference in the participants’ views and attitudes towards their weight and diet between male and female subjects (**Supplementary Tables 3**, **4**).

**Table 3:** Participants’ views and attitudes towards their weight and diet.

Characteristics	T2D Patients	Nondiabetic subjects	OR CI p-value
Weight concern, %: • Not concerned • Little concerned • Moderately or very concerned	41.9 38.8 19.4	42.0 26.1 31.8	0.90 (.49, 1.67).74
Value of diet change, %: • Low • Moderate • High	4.6 78.6 16.8	8.5 67.0 24.5	0.93 (.45, 1.92).84
Self-confidence in diet change, %: • Low • Moderate • High	4.6 80.2 15.3	8.5 67.0 24.5	0.85 (.41, 1.77).66

Ordinal regression analysis upon adjustment for age, gender and BMI. Exposure: patient status

All results did not materially change upon additionally controlling for the participants’ educational status.

## Discussion

This study offers crucial insights into the lifestyle factors that contribute to type 2 diabetes management and prevention within the UAE population. Given the rising prevalence of T2D in the UAE (1, 3, 4), understanding the role of dietary habits, physical activity, and their association with T2D traits is essential for optimizing healthcare interventions. Our finding that individuals with T2D reported healthier dietary habits than their nondiabetic counterparts is particularly noteworthy, as it suggests that diabetes may influence individuals to make healthier food choices, potentially contributing to improved lipid profiles and other metabolic markers. However, the study also highlights a significant disparity in physical activity levels between male diabetic and nondiabetic subjects, with nondiabetic men being significantly more active. This gender-specific difference in physical activity warrants further investigation, as it could have important implications for targeted health promotions and interventions aimed at increasing physical activity among individuals with T2D, particularly in female subjects.

This study was performed in the sample of the UAE nationals residing in Dubai. Although the participants were recruited randomly during the course of this study, the majority of them were overweight or obese, including the patients with T2D and nondiabetic subjects. Thus, these findings indicate an increased prevalence of overweight/obesity in this population sample, which aligns with previous studies demonstrating an increased prevalence of overweight/obesity in the UAE (7, 11, 12, 22), including the Emirate of Dubai (27). The cross-sectional survey performed in the Emirate of Dubai in 2019 (27) reported that the overall prevalence of overweight and obesity was about 40% and 18%, respectively, with the highest obesity rates being in UAE nationals (39.6%) as compared to the other ethnic groups living in this Emirate. It was shown that similar to Kuwait and Saudi Arabia, the UAE has one of the highest prevalence of overweight/obesity (2, 8) and type 2 diabetes (28) among the Gulf Cooperation Council (GCC) countries. Furthermore, a systematic review by Radwan et al. (11) reported a high prevalence rate of obesity and diabetes in the UAE, as well as noticed a 2 to 3-fold increase in the prevalence of overweight/obesity between 1989 and 2017.

A few studies performed in the UAE pointed out the role of gender in obesity risk, with the male individuals appearing to be more prone to being obese than females (17). Another recent study demonstrated an alarming high prevalence of extreme obesity in the UAE, especially among boys (29, 30). On the other hand, previous reports performed in the adult UAE population showed a high percentage of females being overweight (27%) and obese (16%), suggesting that adult females were more prone to be obese than adult males (31). This is in line with recent findings, which also demonstrated that women had a higher BMI across different countries in the Middle East, including the UAE (32). Furthermore, a recent cross-sectional survey performed in the Emirate of Dubai in 2019 reported a high obesity rate (21.6%), as well as a higher obesity risk in female than in male subjects (27). Our results did not show a significant difference in BMI between female and male subjects in this sample of the Emirati population. A reason for this discrepancy might be the smaller sample size in the current study. However, there was a difference in BMI between female diabetic and nondiabetic subjects upon age adjustment, while the significant difference in BMI between T2D and nondiabetic male individuals was not observed.

The risk for becoming overweight and obese has been linked with the urbanization and high socioeconomic status observed in Gulf countries, including Saudi Arabia and the UAE (33, 34). It appears that the influx of Western lifestyles led to changes in behavioral patterns and in the habits of food consumption in the UAE, through having an easy access to processed food and high-calorie beverages, as well as to a sedentary lifestyle (17, 28). It was shown that participants’ fast-food consumption and time spent watching TV were associated with an increased BMI (13), and that consuming less than five servings of fruits/vegetables per day and being physically inactive are the leading predictors of obesity (30). Furthermore, the suboptimal diet was found to be the primary risk factor for cardiometabolic disease mortality in the Middle East countries, accounting for 72% in the UAE (32). Our study showed that the average dietary score was higher in T2D patients than in nondiabetic subjects, suggesting that diabetic patients improved their diet following the diagnosis of diabetes by being more likely to consume less fast food and sweets, and more fruits and vegetables, as indicated by the questionnaire. These adopted changes in their diet probably had a beneficial effect on the lipid profile, including total cholesterol and LDL-cholesterol levels, which were lower in patients with T2D as compared to nondiabetic subjects in this sample of UAE population. Choosing to consume a healthier diet might be related to an increased awareness of diabetic patients participating in our study about the impact of changing their dietary habits on diabetes management. As demonstrated by the questionnaire, altering their diet appeared to be important to all study participants, with the apparent higher percentage of T2D patients considering moderate or high value of diet change vs nondiabetic subjects.

The global age-standardized prevalence of insufficient physical activity is reported to have reached 27.5% in 2016, with the prevalence of insufficient physical activity being particularly increased in the GCC, such as Kuwait (67%), Saudi Arabia (53%), and the UAE (41%) (35). Since most of these countries are based on the service sector, with a low number of physically demanding jobs, this is an additional factor contributing to decreased rates of physical activity observed in the GCC (36). Our results showed that most of the participants (78.8%) are physically inactive. We also demonstrated a trend of lower physical activity in patients with T2D as compared to nondiabetic subjects in this sample of the UAE population. Furthermore, our findings showed that the female participants had lower physical activity than their male counterparts. This is in line with the previous study that demonstrated lower physical activity in women than in men (35). These observations could be related to the cultural and social aspects in the UAE, where boys are found to be more educated about exercise, more encouraged to exercise, seem to have better access to exercise than girls, and appear to comply more with exercise guidelines than girls (28).

There are several limitations of this study. Since it included the adult population from Dubai, our results cannot be applied to the general UAE population. The study sample was restricted to UAE nationals, which means the results may not be applicable to non-UAE residents or other populations with different cultural or lifestyle characteristics. Furthermore, due to the cross-sectional design of this study, we could not establish causality between diabetes diagnosis and dietary habits. However, although we couldn’t establish causality, to the best of our knowledge, this is the first study exploring dietary knowledge, awareness and confidence in diet change in the UAE outpatient population. The study’s relevance to the UAE population is an important strength, as the country is experiencing a growing prevalence of type 2 diabetes, making these findings highly applicable for healthcare professionals and policymakers seeking to address this pressing health concern. Furthermore, this study employed rigorous statistical methods, including linear and logistic regression models, to analyze the data while controlling for important confounding variables such as age, gender, and BMI, ensuring that the findings are not biased by these factors. Another notable strength of the study is its focus on gender-specific analysis, which revealed important differences in physical activity levels between male and female participants and suggested that clinical interventions could be more effective if tailored according to gender. It would be pertinent to increase public health efforts to raise awareness about the role of physical activity in diabetes management, particularly for women, to reduce the overall disease burden and improve quality of life for individuals at risk of or living with T2D. This is in line with the National Nutrition Strategy 2022–2030 launched by the UAE’s Ministry of Health and Prevention (37) and the UAE’s 2021 vision, which pointed at the prevalence of diabetes and the prevalence of obesity in children among the primary national healthcare performance indicators in public healthcare interventions (38). Accordingly, it would be essential to increase awareness about healthy food choices and physical activity, along with developing effective policies and interventions for the nation’s healthy way of life.

Our findings underscore the importance of personalized care strategies that address not only dietary habits but also physical activity levels and gender-specific health behaviors. Healthcare providers in the UAE should further develop clinical and community care practices to enhance preventive measures and design more effective, culturally tailored programs to mitigate the growing burden of T2D. This is in line with the recently developed case management approach (39), where specialized nurses with specific skills in promoting lifestyle changes and managing chronic diseases contribute to the efforts of a multidisciplinary team of healthcare professionals to optimally manage T2D (40). In this particular context, the lifestyle medicine case manager nurse could play a crucial role in T2D management by assisting with their care and offering a tailored care plan, contributing to the overall enhancement of the patient’s clinical condition (41).

In conclusion, our findings demonstrated that majority of the participants in this sample of UAE nationals living in Dubai were obese or overweight. These results are in accordance with the previous studies indicating that the combination of persisting traditional cultural practices, modern cultural changes, and economic prosperity in the Gulf region, including the UAE, created an obesogenic environment that promotes unhealthy eating, sedentary lifestyles, and weight gain, that contributes to the rise in “lifestyle diseases,” such as obesity and T2D (42). Our results showed that the patients with T2D had a lower dietary score than nondiabetic subjects, indicating that they potentially improved their diet following diagnosis by being more likely to consume less fast food and sweets, and more fruits and vegetables. These adopted changes in their diet probably had a beneficial effect on the lipid profile, including total cholesterol and LDL-cholesterol levels, which were decreased in patients with T2D. Furthermore, we also observed a trend of lower physical activity in patients with T2D vs nondiabetic individuals, as well as decreased physical activity in female vs male participants. Understanding dietary habits, physical activity levels, and associated clinical parameters can guide healthcare providers in developing targeted, personalized interventions to improve management and prevention strategies for T2D. The findings suggest that although patients with T2D in the UAE may adopt healthier dietary habits, there remains a significant need to address physical activity disparities, particularly among women, to improve overall health outcomes in this population. Thus, further studies are needed to explore the long-term effects of dietary and physical activity interventions on the health outcomes of T2D patients in the UAE, with particular attention to gender differences and cultural factors influencing lifestyle behaviors.

## Data Availability

The original contributions presented in the study are included in the article/**Supplementary Material**. Further inquiries can be directed to the corresponding author.
