# Supplementary material for: Lifestyle habits and type 2 diabetes traits in patients from healthcare centers in Dubai, United Arab Emirates: a cross-sectional study
Source: Front Endocrinol (Lausanne). 2025 May 6;16:1436536. doi: 10.3389/fendo.2025.1436536 (PMC12088936; doi:10.3389/fendo.2025.1436536)
Supplement: Supplementary file 1 [file DataSheet1.docx]

**Supplementary Table 1.** The clinical and biochemical parameters in T2D patients and nondiabetic **male** subjects.

|  | **Model I*** | | | **Model II**** | | |
| --- | --- | --- | --- | --- | --- | --- |
| **Parameters** | **B** | **CI** | **p-value** | **B** | **CI** | **p-value** |
| Systolic, mmHg | -1.0 | (-6.7, 4.6) | .7 | -1.8 | (-7.2, 3.7) | .5 |
| Diastolic, mmHg | -3.2 | (-7.6, 1.3) | .1 | -3.7 | (-8.0, .7) | .1 |
| BMI, kg/m3 | 1.1 | (-1.4, 3.7) | .4 | - | - | - |
| Fasting glucose, mmol/L | 2.5 | (1.1, 3.9) | **<.001** | 2.5 | (1.1, 3.9) | **<.001** |
| Fasting insulin, pmol/L | 40.7 | (-31.5, 112.9) | .3 | 39.9 | (-26.8, 106.7) | .2 |
| HOMA-IR | 6.1 | (-3.7, 15.8) | .2 | 5.6 | (-3.8, 15.1) | .2 |
| HbA1C, mmol/mol | 19.2 | (12.2, 26.2) | **<.001** | 19.0 | (12.0, 26.0) | **<.001** |
| Total cholesterol, mmol/L | -1.5 | (-2.6, -.4) | **.009** | -1.5 | (-2.6, -.4) | **.01** |
| Triglyceride, mmol/L | 1.9 | (.5, 3.3) | **.007** | 1.9 | (.5, 3.3) | **.008** |
| HDL-cholesterol, mmol/L | -.4 | (-.7, -.1) | **.006** | -.4 | (-.7, -.1) | **.008** |
| LDL-cholesterol, mmol/L | -1.4 | (-2.4, -.4) | **.009** | -1.4 | (-2.5, .4) | **.009** |
| CRP, mg/dL | 2.3 | (-5.0, 9.6) | .4 | 2.0 | (-5.5, 9.4) | .6 |

********Linear regression analysis prior to adjustment for age;* ******** *Linear regression analysis upon adjustment for* *age and BMI.*

**Supplementary Table 2.** The clinical and biochemical parameters in T2D patients and nondiabetic **female** subjects.

|  | **Model I*** | | | **Model II**** | | |
| --- | --- | --- | --- | --- | --- | --- |
| **Parameters** | **B** | **CI** | **p-value** | **B** | **CI** | **p-value** |
| Systolic, mmHg | 3.5 | (-1.6, 8.6) | .2 | 2.1 | (-2.9, 7.0) | .4 |
| Diastolic, mmHg | -1.3 | (-4.9, 2.3) | .5 | -1.7 | (-5.3, 2.0) | .4 |
| BMI, kg/m3 | 2.3 | (.1, 4.4) | **.04** | **-** | **-** | **-** |
| Fasting glucose, mmol/L | 1.7 | (.9, 2.5) | **<.001** | 1.6 | (.7, 2.4) | **<.001** |
| Fasting insulin, pmol/L | 29.6 | (7.7, 51.5) | **.009** | 25.6 | (3.6, 47.7) | **.023** |
| HOMA-IR | 1.8 | (.7, 2.9) | **.002** | 1.6 | (.5, 2.7) | **.006** |
| HbA1C, mmol/mol | 11.9 | (6.8, 17.0) | **<.001** | 11.1 | (5.9, 16.2) | **<.001** |
| Total cholesterol, mmol/L | -1.5 | (-2.3, -.7) | **<.001** | -1.5 | (-2.3, -.7) | **<.001** |
| Triglyceride, mmol/L | .9 | (-.05, 1.8) | .06 | .8 | (-.1, 1.7) | .09 |
| HDL-cholesterol, mmol/L | -.5 | (-.7, -.2) | **.003** | -.5 | (-.7, -.2) | **<.001** |
| LDL-cholesterol, mmol/L | -1.2 | (-1.9, -.5) | **<.001** | -1.2 | (-1.9, -.5) | **<.001** |
| CRP, mg/dL | 2.6 | (-10.8, 16.0) | .7 | 2.1 | (-11.4, 15.5) | .8 |

********Linear regression analysis prior to adjustment for age********* *Linear regression analysis upon adjustment for* *age and BMI.*

**Supplementary Table 3.** **Male** participants’ views and attitudes towards their weight and diet.

| **Characteristics** | **T2D Patients** | **Nondiabetic subjects** | **OR CI p-value** |
| --- | --- | --- | --- |
| Weight concern, %:   - Not concerned - Little concerned - Moderately or very concerned | 41.9  38.8  19.4 | 42.0  26.1  31.8 | 0.99 (.31, 3.21) .98 |
| Value of diet change, %:   - Low - Moderate - High | 4.6  78.6  16.8 | 8.5  67.0  24.5 | 0.68 (.18, 2.61) .58 |
| Self-confidence in diet change, %:   - Low - Moderate - High | 4.6  80.2  15.3 | 8.5  67.0  24.5 | 0.62 (.17, 2.29) .47 |

*Ordinal regression analysis upon adjustment for* *age and BMI.*

**Supplementary Table 4.** **Female** participants’ views and attitudes towards their weight and diet.

| **Characteristics** | **T2D Patients** | **Nondiabetic subjects** | **OR CI p-value** |
| --- | --- | --- | --- |
| Weight concern, %:   - Not concerned - Little concerned - Moderately or very concerned | 41.9  38.8  19.4 | 42.0  26.1  31.8 | 0.87 (,42, 1.80) .70 |
| Value of diet change, %:   - Low - Moderate - High | 4.6  78.6  16.8 | 8.5  67.0  24.5 | 1.04 (.44, 2.51) .91 |
| Self-confidence in diet change, %:   - Low - Moderate - High | 4.6  80.2  15.3 | 8.5  67.0  24.5 | 0.98 (.40, 2.39) .97 |

*Ordinal regression analysis upon adjustment for* *age and BMI.*
